# An 8-Week Peer Health Coaching Intervention among College Students: A Pilot Randomized Study

**DOI:** 10.3390/nu15051284

**Published:** 2023-03-04

**Authors:** Zi Yan, Jessica Peacock, Juliana F. W. Cohen, Laura Kurdziel, Sarah Benes, Seungbin Oh, April Bowling

**Affiliations:** 1 Department of Public Health and Nutrition, School of Nursing and Health Sciences, Merrimack College, North Andover, MA 01845, USA; 2 Department of Exercise Sciences and Rehabilitation, School of Nursing and Health Sciences, Merrimack College, North Andover, MA 01845, USA; 3 Department of Psychology and Neuroscience, Merrimack College, North Andover, MA 01845, USA; 4 Department of Health and Movement Science, Southern Connecticut State University, New Haven, CT 06515, USA; 5 Mental Health Counseling & Behavioral Medicine Program, Department of Psychiatry and Graduate Medical Sciences, Boston University School of Medicine, Boston, MA 02118, USA; 6 Department of Psychiatry, University of Massachusetts TH Chan Medical School, 55 N Lake Ave., Worcester, MA 01655, USA

**Keywords:** diet, mental health, physical activity, positive affect, sleep, stress

## Abstract

This study explored the effects of an 8-week peer coaching program on physical activity (PA), diet, sleep, social isolation, and mental health among college students in the United States. A total of 52 college students were recruited and randomized to the coaching (n = 28) or the control group (n = 24). The coaching group met with a trained peer health coach once a week for 8 weeks focusing on self-selected wellness domains. Coaching techniques included reflective listening, motivational interviews, and goal setting. The control group received a wellness handbook. PA, self-efficacy for eating healthy foods, quality of sleep, social isolation, positive affect and well-being, anxiety, and cognitive function were measured. No interaction effects between time and group were significant for the overall intervention group (all *p* > 0.05), while the main effects of group difference on moderate PA and total PA were significant (*p* < 0.05). Goal-specific analysis showed that, compared to the control group, those who had a PA goal significantly increased vigorous PA Metabolic Equivalent of Task (METs) (*p* < 0.05). The vigorous METs for the PA goal group increased from 1013.33 (SD = 1055.12) to 1578.67 (SD = 1354.09); the control group decreased from 1012.94 (SD = 1322.943) to 682.11 (SD = 754.89); having a stress goal significantly predicted a higher post-coaching positive affect and well-being, controlling the pre-score and other demographic factors: *B* = 0.37 and *p* < 0.05. Peer coaching showed a promising effect on improving PA and positive affect and well-being among college students.

## 1. Introduction

According to the Centers for Disease Control and Prevention [1], approximately one in two people in the United States have at least one chronic health condition, such as heart disease, cancer, hypertension, diabetes, or obesity. One in four adults have two or more chronic health conditions. It has been estimated that a major portion of chronic conditions could be prevented by behavior-related lifestyle interventions [1]. 

College is a critical lifetime transition period for young adults to develop and independently practice healthy behaviors [2]. Poor health behavior practices and associated mental health challenges have emerged as critical risks among undergraduate students in the United States [3]. College students are at risk of gaining weight due to a lack of physical activity (PA), poor nutrition [4], and lack of sleep [5]. Approximately 25% of college students suffer from mental health challenges, such as anxiety, depression, and alcohol use disorder [6]. 

The previous literature has documented the evidence of lifestyle interventions as a public health strategy to prevent and manage chronic illness [7], as well as improve well-being [8]. Among various lifestyle interventions, health coaching holds promise to promote healthy behavior practices due to its ability to address multiple behaviors, health risks, and the self-management of illness in a cost-effective manner [9]. Health coaches foster individuals’ autonomy and intrinsic motivations [10,11], as well as provide support for the intrapersonal process that “energizes and directs behaviors towards healthier and more successful human functioning” [12]. Although there is a lack of agreement on which theory of health coaching is most prominently grounded, it is suggested that the strategies used in health coaching, including listening, asking powerful questions, and motivational interviewing, are consistent with constructs of the self-determination theory (SDT) [13]. According to the SDT, when health coaches provide an environment and guidance to facilitate individuals’ competence, autonomy, and relatedness, they will be intrinsically motivated to set health-related goals and work towards their best health status and human function [12,14]. 

The use of health coaching has become widespread in recent years and its effectiveness in promoting health behavior practices has been well supported by the available evidence [15,16]. Health coaching studies have shown significant improvements in various health outcomes, including weight loss and management, improved mental health, and enhanced performance of activities of daily living for various populations, including adults with fibromyalgia, eating disorders, and heart diseases [17,18]. 

Colleges and universities are ideal settings to improve well-being among young adults. Behavioral interventions in college settings have the potential to reach a larger group of young adults. Colleges and universities also provide a unique opportunity for students with health-related majors to be trained and serve as the health coaches for their peers. Peer support is reported to be particularly important in health promotion programs among youth and young adults [19]. Living in a similar physical and social environment allows the student coaches to better understand the experience and challenges of the individuals they coach. In addition, being of similar ages and sociocultural backgrounds may facilitate rapport in the coaching relationship. Previous peer education studies also showed that student coaches improved cultural competence and health behavior practices after participating in peer health promotion programs [20,21]. 

Previous health coaching studies have primarily focused on physiological outcomes (i.e., blood pressure, HbA1c, blood glucose, cholesterol, BMI, or body weight), while behavioral outcomes were often disregarded [22]. Those health coaching studies that evaluated behavior outcomes often had pre-decided wellness topics such as PA or nutrition [23,24] on which coaching would be focused, regardless of what health topics may be of importance to those being coached. While this approach can more easily quantify the dosage of the intervention on the specific health or wellness topic, it restricts participants’ autonomy over wellness topics. Restricting specific wellness topics also limits the ability to detect the potential interaction among different health behaviors. For example, research has shown that increased PA is also associated with better sleep quality, as well as improved mental health [25,26,27]. Treating a higher-order construct, such as motivation, may link treatment to lower-order constructs, such as specific behaviors. Thus, it is possible that the clients who worked on improving one aspect of wellness through coaching may have other behavioral benefits [28]. 

In addition, prior health coaching studies have primarily targeted participants with certain types of disease or chronic conditions, such as cancers, diabetes, obesity, cardiovascular disease, and pulmonary diseases [29,30,31], with limited studies on healthy populations such as college students. For the handful of health coaching programs that targeted healthy college students, none of them have used randomized designs (RCTs) [32,33,34]. In a recent review study, An and colleagues noted that very few RCTs have evaluated the effects of health coaching and called for more RCTs of the health coaching studies [22].

In response to the nationwide call for more community- and evidence-based programs to improve population health, the research team designed this pilot program to gather scientific evidence to evaluate the effectiveness of the peer health coaching program in the college campus setting. Therefore, the current study aimed to assess the effectiveness of a randomized, 8-week peer health coaching program on PA, nutrition, sleep, social isolation, and mental health, among college students. 

## 2. Materials and Methods

### 2.1. Study Design

The study used a randomized, 8-week interventional design to evaluate the efficacy of a peer health coaching intervention delivered in a college setting. The study was conducted at a midsize private college (i.e., student population ~4000 to 5000 students) located in New England, USA. The baseline and post-intervention assessment were conducted in January 2022 and May 2022, respectively. The Institutional Review Board (IRB) at the college approved all study procedures. 

### 2.2. Participants

Student participants. Freshmen and sophomore students 18 years or older were eligible to participate. Participants were recruited through campus flyers, recruitment tables in front of the student center building, and classroom visits to a course that all freshmen are required to take. We also purposely recruited from programs serving historically underrepresented populations, including first-generation students and students from minoritized races/ethnicities, since such students were disproportionately affected by the pandemic and generally experienced greater barriers to participation in health programming. A total of 52 participants were recruited. After completing the pre-assessment, they were randomly assigned into the coaching group (n = 28) or the control group (n = 24). 

### 2.3. Intervention Protocol

#### 2.3.1. Health Coaches

Student coaches were health science major undergraduate students who were enrolled in a series of two health coaching courses that prepared them for basic coaching skills and improving skills and self-efficacy through practice. The courses were taught by a group of faculty. Two of those faculty have received WellCoaches^®^ health coaching certification; other faculty had related expertise (e.g., cultural studies, counseling, mental health). In the first coaching course, students had received the basic training on coaching theories and techniques. They all passed a mock health interview exam by the end of the first coaching course and before they started the second coaching courses, in which they needed to complete 50 coaching sessions. This study was conducted as part of their 50-session training. During this study and throughout the course, student coaches met with the course instructor and other coaches weekly to discuss their coaching progress and challenges. In addition, each coach met with the course instructor individually every other week to receive additional feedback and support. 

#### 2.3.2. Procedures

**Figure A1:**
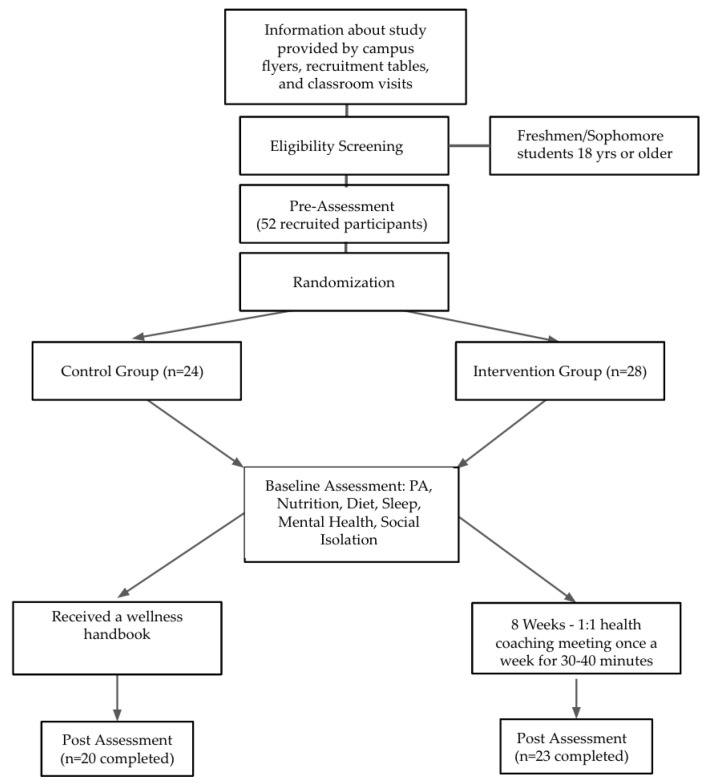
Intervention flowchart.

After completing the baseline assessment, participants were randomized to either the intervention (i.e., coaching group) or the control group. Covariate adaptive randomization was adopted to balance the participants between the intervention and control groups on gender, first-year students, and students of color. Participants in the coaching group met with their assigned 1:1 peer health coach once a week for 8 weeks. Coaching meetings were scheduled for 30–40 min. Coaching meetings were in-person but zoom coaching meetings were allowed if students were sick or had safety concerns related to COVID-19. See Appendix Figure A1 for the flow chart of the intervention.

#### 2.3.3. Intervention

The health coaching program was designed to facilitate and emphasize several aspects. The first coaching session focused on self-goal identification. At the first coaching session, the health coaches assisted the students to identify 2–3 areas within the topics of PA, nutrition, sleep, and social support that they would like to improve on. During each coaching meeting, student coaches evaluated their previous weekly goals and discussed their gains and challenges and areas that they would like to work on in the coming weeks. Second was “Peer support”. In each session, coaches facilitated the discussion using coaching techniques, including reflective listening, affirmation, motivational interviewing, etc. Lastly, the health coaching program facilitated and emphasized “Goal setting.” By the end of each session, coaches assisted student participants to come up with two SMART goals (i.e., specific, measurable, action-based, realistic, time-limited) that they felt ready to work on. Notably, participants were encouraged to set behavioral goals that they would like to work on. That said, no specific behavior goal was pre-set for participants. For instance, one participant may set a weekly physical activity goal of walking more, whereas another participant may work on doing more moderate-vigorous exercise. In the following week, students then conducted a self-evaluation on the completion rate from 0 percent to 100 percent on each of the wellness goals. 

Students in the control group received a wellness handbook that was created by the research team. This handbook provided information related to how to improve physical activity, nutrition, sleep, stress, and social support in the college setting. Students in the control group were asked to use this handbook as an information source if they would like to improve their health behavior practices.

### 2.4. Measures

#### 2.4.1. Demographics

Gender, age, race, and whether they were first-generation students or student athletes were collected. In addition, socioeconomic status was measured by the MacArthur Scale of Subjective Social Status [35]. Students indicated their perception of their social status by rating a 10-point Likert-type scale from 1 (lowest standing in the community) to 10 (highest standing in their community). Previous studies indicated good evidence of reliability and validity on scores of the measure [36,37]. 

#### 2.4.2. Physical Activity (PA)

The International Physical Activity Questionnaire (IPAQ)—short form was used as the subjective measure of physical activity. A total of 7 items were structured to provide separate METs (min/week) on walking; moderate-intensity activity; vigorous-intensity activity, total physical activity, and time spent on sitting (walking MET—minutes/week = 3.3 × walking minutes × walking days; moderate MET—minutes/week = 4.0 × moderate-intensity activity minutes × moderate days; vigorous MET—minutes/week = 8.0 × vigorous-intensity activity minutes × vigorous-intensity days). This questionnaire has demonstrated acceptable reliability and validity on the total score in previous studies [38]. Exercise self-efficacy was also assessed using a five-item measure to assess the confidence to perform physical activity in five different situations (i.e., vacation, feeling tired, bad mood, not having enough time, and bad weather [39]. This measure has been shown to predict physical activity in previous studies [40].

#### 2.4.3. Diet

Healthy eating self-efficacy was assessed as a summary of nine five-point Likert-scaled questions about self-confidence for eating healthy foods at the mall, after school, with friends, under stress, feeling down, bored, at a fast-food restaurant, alone, and at family dinner (not confident at all = 1 to very confident = 6; (Cronbach *a* = 0.83)) [41].

#### 2.4.4. Sleep

The Pittsburgh Sleep Quality Index (PSQI) [42] was used as a subjective measure of sleep quality. It differentiates “poor” from “good” sleep quality by measuring seven areas (components): subjective sleep quality, sleep latency, sleep duration, habitual sleep efficiency, sleep disturbances, use of sleeping medications, and daytime dysfunction over the last month. A Global PSQI score was calculated by adding scores from the 7 areas together. A total score of 5 or higher indicates a poor sleep quality. 

#### 2.4.5. Social Isolation

NIH Item bank V2.0 Social Isolation (Short Form) from PROMIS was used to measure social isolation. Participants responded to a four-item scale. An example question was: “In the last 7 days, how often do you feel (e.g., I felt left out).” Responses ranged from Never (1) to Always (5). The sum score ranged from 4 to 20, with a higher score indicating higher social isolation.

#### 2.4.6. Mental Health

For the purposes of this study, we utilized validated measures developed as part of the NIH-funded Patient-Reported Outcomes Measurement Information System (PROMIS). Positive affect and well-being were measured using the Neuro-QOL Item Bank v1.0—Positive Affect and Well-Being (Short Form). An example question was “Lately, my life was satisfying…” Responses range from Never (1) to Always (5). The instrument is 9 items with a score range of 9 to 45, where higher scores indicated a higher overall positive affect and well-being. Anxiety was measured by the Neuro-QOL Item Bank v1.0—Anxiety (Short Form) from the PROMIS. An example question was “In the past 7 days, I felt nervous.” Responses ranged from Never (1) to Always (5). The instrument features 8 items with a score range of 8 to 40. A higher score indicates a higher anxiety level. Cognitive function was measured by NIH Neuro-QOL Item Bank v2.0—Cognition Function (Short Form) from the PROMIS. Four questions started with “In the past 7 days… (e.g., I had to read something several times to understand it).” Answering options ranged from “Never (5)” to “Very often (several times a day) (1).” Another four questions started with “How much DIFFICULTY do you currently have… (e.g., learning new tasks or instructions?)”, and answering options range from “None (5)” to “Cannot do (1).” The total score ranged from 8 to 40, with higher scores indicating better cognitive function.

### 2.5. Data Analyses

Intention-to-treat analysis was performed. In addition to descriptive statistics, repeated MANOVA was used to explore the overall impact of the coaching intervention on physical activity, sleep, nutrition, mental health, and social isolation between participants in the coaching group and the control group. In addition, participants in the coaching group were further divided into subgroups based on the wellness goals they identified. Repeated ANOVA and MANOVA analyses were used to explore the coaching effect on the goal-specific domain. For instance, participants who identified physical activity as one of their coaching goals were compared to the control group on the physical activity variables. Regression analysis was performed to examine the effects of the demographic factors on the post-assessment scores, controlling the pre-assessment scores. All data were analyzed using SPSS 21 (IBM, Armonk, New York, NY, USA).

## 3. Results

Demographics: Two participants (7%) in the coaching group (n = 28) dropped the study during the intervention. Among the rest of the 26 students who completed the 8-week intervention, 23 (82%) completed the post-assessment. For the remaining 24 participants in the control group, 20 (83%) of them completed the post-assessment. Table 1 also showed that there was no significant difference in the demographic characteristics between the coaching and control groups.

*Intervention Effect Analysis*. Means and standard deviations for all variables are shown in Table 2. No variables were significantly different at baseline between intervention and control groups. All scales demonstrated moderate to good reliability, with Cronbach’s alpha values ranging from 0.80 to 0.95. Repeated measures MANOVA tests showed that all the interaction effects between time and group were not significant, all *p* values > 0.05; the main effects of group difference on Moderate PA MET and total PA MET were significant: *F*(1,39) = 4.76, *p* = 0.035, *F*(1,39) = 4.99, and *p* = 0.031, respectively. The vigorous PA MET was marginally significant: *F*(1,39) = 4.06 and *p* = 0.051. The main effects of time and group were not significant for all other variables, all *p* values > 0.05.

### Goal-Specific Analysis

Physical Activity. A total of 15 participants in the coaching group (65%) identified PA as their coaching goals and discussed PA-related goals for at least one coaching meeting. The average goal meeting rate was 73%.

Repeated MANOVA results showed that the interaction effects between time and group on Vigorous MET was significant *F*(1,32) = 6.42, *p* = 0.017. For those who identified PA as one of their coaching goals, vigorous MET increased from 1013.33 (1055.12) to 1578.67 (1354.09), while the control group decreased from 1012.94 (1322.94) to 682.11 (754.89). All other interaction and main effects were not significant, all *p* values> 0.05. 

We also explored whether having PA as a coaching goal, as well as the demographic factors, would predict more PA changes. The regression analyses showed that, controlling for pre-PA level and the demographic factors, coaches who had PA as a coaching goal had a marginal significant higher post-total PA MET (*B* = 0.35, *p* = 0.053) and a significant higher post-vigorous PA MET (*B* = 0.44, *p* < 0.01) than those who did not have PA as a coaching goal and those who were in the control group. See Table 3 and Table 4. 

*Diet.* Fifteen students (65%) in the coaching group identified improving diet as one of their coaching goals. The overall self-evaluation goal completion rate was 87%. The interaction between group and intervention as well as the main effect of group and time were not significant: *F*(2,39) = 0.01, *F*(1,39) = 0.41, and *F*(1,39) = 0.76, respectively, all *p* values > 0.05. The Healthy eating efficacy for the goal-specific group changed from 27.4 (8.51) to 28.2 (7.03), compared to from 25.68 (5.73) to 26.47 (5.32) for the control group. Having diet as a coaching goal, as well as other demographic factors, did not predict the healthy eating efficacy score. See Table 5 for details.

Sleep. A total of 13 participants in the coaching group (57%) identified sleep as one of their coaching goals, with the goal completion rate of 79.96%. The interaction between group and intervention was not significant: *F*(1,28) = 3.60 and *p* > 0.05. The group differences were significant: *F*(1,28) = 5.18 and *p* < 0.05. Among the 13 participants who identified sleep as one of their coaching goals, the average Global PSQI score decreased from 6.18 (2.35) in the pre-test to 4.36 (2.20) in the post-test; as for the control group, the score changed from 7.74 (2.76) to 7.26 (3.58). Regression analysis showed no significant intervention effect on predicting post-PSQI scores. See Table 5 for the details.

Social Isolation. Seven students (30%) identified improving social-related support as one of their coaching goals, with the self-evaluated goal completion rate of 75%. There were no significant intervention or group effects, all *p* values > 0.05. The social isolation score of the intervention group changed from 9.33 (5.84) to 10.33 (6.12), while the control group changed from 9.74 (3.79) to 9.15 (3.67) from the pre- and post-assessment. Compared to those in the control group, having the social goal as a coaching goal did not predict the post-social isolation score; however, females had significantly less social isolation than males, controlling all other variables: B = −0.42 and *p* < 0.01.

Mental Health. Although no student directly identified “mental health” as one of the coaching goals, seven participants in the coaching group identified stress management as one of their coaching goals (30%) and discussed stress-related goals during at least one coaching session. The overall self-evaluated goal completion rate was 83.09%. The interactions between group and intervention on anxiety, positive affect and well-being, and cognitive function were not significant, all *p* values > 0.05. The main effect of the group was significant on anxiety (*F*(1,24) = 5.18, *p* < 0.05). The anxiety score for the goal-specific group changed from 14.14 (6.04) to 14.71 (7.18), while for the control group it changed from 22.05 (7.82) to 20.52 (7.29). All other main effects were not statistically significant. Compared to those who were in the control group, those who had a stress goal had a significantly higher post-positive affect and well-being score, controlling pre-positive affect score and other demographic factors, B = 0.37, *p* < 0.05. No relationships were identified between a stress goal and post-anxiety and cognitive function.

## 4. Discussion

Although the concept of a health coach and peer education model has been well developed, there has been very little empirical research on the effectiveness of peer health coaching programs. Even less is known regarding the effectiveness of health coaching among non-clinical young adult populations in the higher education setting. To the authors’ knowledge, this is the first published study in which multiple health behavior outcomes were evaluated via a health coaching RCT among college students. 

The most important findings were that the students in the coaching group who worked on PA showed significant intervention effects on vigorous PA and marginally significant improvement on total PA over the 8-week period. This is consistent from previous studies. For instance, a meta-analysis with 27 randomized trials shows a small, significant effect size (SMD = 0.27) in PA improvement achieved by health coaching among people aged 60 years or older [43]. In another meta-analysis study [22] that examined health coaching intervention among adults with cardiovascular disease, the effect size of health coaching intervention is small (effect size < 0.20) for physical activity and diet. The current study provided preliminary evidence of the effectiveness of health coaching on physical activity among healthy young adults. 

Although there was no significant intervention effect on walking and moderate PA, the improvement of vigorous PA and marginally significant improvement on total PA among the participants indicated that they engaged in more planned exercise behaviors after the intervention. Interestingly, while vigorous and total PA increased among the participants, there was a decrease in vigorous and total PA among the control group. This may be due to the fact that post-assessment was one week before the final examinations, and the increased stress and study time may have made students in the control group less likely to engage in PA [44]. This also demonstrates that peer health coaching not only buffered the negative impact of increased stress and lack of time, but also provided additional support for students to engage in PA during a challenging time. 

We also found that individuals who worked on stress-management-related goals had an improved positive affect and well-being, compared to those in the control group. This is supported by the previous research indicating that stress evokes a negative affect [45] and successful stress management could improve an individual’s positive affect [46]. In addition to discussing stress-management-related issues, previous research also suggested that the peer support provided by the health coaches may play a role in improving participants’ positive affects [47]. Future path analysis and qualitative studies may provide additional evidence for this relationship. 

Another strength of the study was that it provided the participants with the antonym to self-select the wellness topics and behavioral goals, and the health coaches were in the supporting role in the coaching relationship. This is different from other health coaching intervention studies that involved adults with health conditions (e.g., diabetes, cardiovascular diseases) in which the topics and behavioral goals were often pre-determined, and health outcomes (vs. behavioral outcomes) were often assessed [11]. Considering that our participants were healthy young adults, they usually did not have health conditions that required them to achieve specific behavioral outcomes. In this case, providing autonomy and peer support would invoke their internal motivation [14,48]. This approach may make the assessment of implementation fidelity more difficult, but it has gained empirical value by supporting participants with on-going, real-world problems. For example, while final exams were approaching towards the end of the study, more student participants chose to work on stress-management-related topics with their coaches. Although there is no direct evidence from the current study, it is possible that the improved stress management skills may benefit other health behavioral practices for the participants. In addition, more flexibility in coaching topics provides practical value for colleges and universities who may consider adopting peer coaching programs in future.

Although not statistically significant, there was a trend towards the improvement of sleep quality among participants who worked on sleep-related goals, compared to the control group. Type II error due to the small sample size may play a role here. No intervention effect was detected among participants who worked on diet-related goals. This may be because, compared to other behavioral goals, diet-related goals were very diverse, from eating breakfast to drinking more water. This makes the intervention effect very challenging to measure without significant noise. We suggest future studies consider personalized diet measures based on the specific diet goals.

Given that first-year students, students of color, students from low-income families, and first-generation students are often disproportionately affected by the pandemic and have a more difficult time transitioning to the academic and social demands of college [49], we purposely recruited students from those populations with success; over 23% of the students were first-generation and 37% were non-white, as compared to 20% and 16% in the college student body as a whole. There were no significant differences in intervention uptake or outcomes by demographic group; however, additional adaptations could be made to the health coaching approach to improve engagement among specific groups and potentially decrease health disparities disproportionately experienced by historically marginalized and under-represented student groups. We suggest future studies continue to explore the potential moderating or mediating effect of demographic factors on health coaching engagement and effectiveness.

There were several limitations of this study. First, the study began in January and ended in May 2022, during which seasonality became a challenge for the participants. That is, the end-of-semester stress may cause college students to have increased stress, worse nutrition, less PA, and poor sleep quality [44,49]. Second, although having student participants choose the health topics that they were interested in provided them with autonomy, working on several topics may have lowered the intervention dosage they received for each topic. For example, participants who chose to work on PA, sleep, and stress had less exposure to each of those three topics than those who only worked on PA or sleep throughout the intervention period. This type of study design also imposes challenges on statistical analyses as it is impossible to know the exact topics that participants would work on. Furthermore, when clients chose the coaching topic of their interest, it may have posed selection bias as they were more motivated to work on this topic than participants in the control groups. Future studies may consider screening participants’ well-being interest and then place them into different coaching groups. In addition, we did not perform a priori power analysis due to limited research on this topic and the pragmatic constraint of the limited trained coaches we had with whom to conduct the study. However, our power, calculated based on the number of participants enrolled, was 50% to detect a 0.5 effect size. Although this is much lower than the ideal power of 80%, we still generated some significant results, indicating a trend towards effectiveness that could be fully ascertained with a full-effectiveness trial. Finally, the current study did not follow up on the participants’ post-intervention. The fidelity of health coaching intervention should be further explored in future studies.

The current study was conducted in a college campus in which intervention was delivered by students majoring in health science who went through training via two health coaching courses. Although they had limited coaching experiences compared to the professional health coaches, they provided unique peer support that other professional coaches would not be able to provide. This study also set an example of an innovative health promotion program in the higher education setting. Additionally, from the education experiential learning perspective, the peer health coaching programs also served as an education and practice opportunity for students with related majors. The experience and skills they developed through this intervention will further prepare them for their future career, especially those who will work in client-facing settings. 

## 5. Conclusions

The post-COVID-19 era poses new challenges for colleges to think creatively to assist young adults with their overall well-being [50,51]. The current pilot study was set in a real-world setting where college students chose their health coaching topics and received support from their peers. It provided important empirical evidence on the effectiveness of peer health coaching in the college setting. That is, an 8-week peer health coaching intervention in a college setting showed preliminary evidence of improving PA and positive affect and well-being for participants who had worked on those topics with their peer health coaches. With more evidence from future studies that are conducted with larger sample sizes and more rigorous methods, the peer health coaching approach has the promise to be scalable and feasible to promote health and well-being among students at institutions of higher education. In addition, colleges and universities may consider adopting the peer health coaching model as an education and training opportunity for health sciences students or those in related majors, such as human development and psychology.

## Figures and Tables

**Table 1:** Participants’ Demographic Characteristics and Completion Rate.

	Coaching Group	Control Group	Group Difference, *p* Value
Number of participants	28	24	
Gender (female)	22 (78.6%)	20 (83.3%)	0.63
Age	18.7 (±7.2)	18.8 (±7.0)	0.51
Race (white)	17 (60.7%)	16 (16.6%)	0.23
First-generation student	7 (25.0%)	16 (66.7%)	0.70
Student athlete	8 (28.6%)	7 (29.2%)	0.94
Socioeconomic status (SES)	5.8 (±1.4)	6.0 (±1.4)	0.79
Withdrawn or lost to follow-up	2	4	0.28
Completed post-assessment	23 (78.6%)	20 (83.3%)	0.26

Note. The number in the () is percentage if it is a count or standard deviation if it is a continuous number.

**Table 2:** Descriptive results of the primary and secondary constructs between coaching and control groups.

		Coaching Group (n = 23)			Control Group (n = 20)		
		Pre	Post	Cohen’s	Pre	Post	Cohen’s
PA	Total PA METs	3052.0 (233.57)	2816.98 (1803.73)	0.43	2050.34 (1589.69)	1813.76 (1003.89)	0.41
	Vigorous METs	1561.74 (1378.24)	1496.53 (1253.89)	0.04	1012.63 (1530.61)	682.11 (754.89)	0.30
	Moderate METs	813.04 (1274.87)	602.61 (634.58)	0.15	342.10 (464.70)	357.89 (433.58)	−0.03
	Walk METs	677.21 (660.21)	674.25 (499.64)	0.03	695.60 (630.61)	773.76 (660.57)	−0.14
	Exercise self-efficacy	14.96 (3.14)	14.87 (3.72)	0.02	13.74 (5.09)	14.42 (4.41	−0.21
Diet	Healthy eating efficacy	27.35 (7.02)	28.04 (6.41)	−0.13	25.68 (5.74)	26.47 (5.33)	−0.19
Sleep	Global PSQI (sleep)	6.43 (2.33)	6.17 (3.63)	0.08	7.74 (2.77)	7.26 (3.59)	0.13
Social isolation	Social isolation	7.48 (4.10)	7.47 (3.88)	0.00	9.74 (3.80)	9.16 (3.67)	0.20
Mental health	Anxiety	18.87 (7.33)	18.43 (8.25)	0.06	22.05 (7.83)	20.53 (7.30)	0.29
	Positive affect	35.43 (7.90)	37.34 (5.18)	−0.32	34.00 (7.82)	33.74 (6.85)	0.05
	Cognitive function	28.39 (5.30)	28.43 (8.41)	−0.01	27.58 (6.85)	29.05 (6.49)	−0.32

Note. METs stands for the average METs per week. The numbers in the () are standard deviations.

**Table 3:** Pre and post PA results between the PA goal group and the control group.

	Coaching Group (n = 15)		Control Group (n = 20)	
	Pre	Post	Pre	Post
Vigorous METs	1013.33 (1055.12)	1578.67 (1354.09) *	1012.94 (1322.94)	682.11 (754.89)
Moderate METs	662.66 (1433.01)	596.00 (552.40)	342.10 (464.70)	357.89 (433.58)
Walking METs	743.60 (721.68)	695.60 (630.61)	729.30 (519.39)	773.76 (660.57)
Total PA METs	2419.60 (2425.32)	2903.07 (1763.69)	2050.34 (1589.69)	1813.76 (1003.89)
PA self-efficacy	14.47 (3.27)	14.20 (3.89)	13.74 (5.08)	14.42 (4.41)

Note. METs stands for the average METs per week. The numbers in the () are standard deviations. * Indicates *p* < 0.05 for the interaction between time and group.

**Table 4:** Regression analysis on post-PA parameters (n = 15).

	Total Post-PA MET	Vigorous PA MET	Moderate PA MET	Walking PA MET
No PA goal	0.07	0.11	0.20	−0.14
PA goal	0.35 ~	0.44 **	0.19	−0.05
Pre-PA	0.41 *	0.52 **	0.15	0.33 *
Female	−0.05	−0.06	−0.03	0.02
FGCS	0.10	0.14	−0.05	0.18
SES	0.18	0.05	0.31	0.02
Race: Black	0.19	0.13	0.03	0.27
Race: Asian	0.02	0.03	−0.06	0.23
Race: biracial	−0.15	−0.27	−0.10	0.16
Race: other	−0.03	−0.09	0.20	−0.15
R2	32.6%	42.4%	18%	42.4%

~ indicates a marginal significance, *p* = 0.053; * indicates *p* < 0.05; ** indicates *p* < 0.01; FGCS = first-generation college students; reference group: control group, male, non-FGCS, white. Standardized coefficients were reported.

**Table 5:** Regression analysis on post-coaching parameters of diet, sleep, social isolation, and mental health.

	Healthy Eating Efficacy (n = 15)	Global PSQI (n = 13)	Social Isolation (n = 7)	Anxiety (n = 7)	Positive Affect (n = 7)	Cognitive Function (n = 7)
No related goal	0.02	1.75	−0.14	−0.01	0.13	−0.18
Related goal	0.03	−1.63	−0.02	−0.08	0.37 *	0.03
Pre-score	0.69 ***	2.72 *	0.64 ***	0.63 ***	0.71 ***	0.45 **
Female	0.08	−0.38	−0.42 **	−0.13	0.01	0.09
FGCS	0.11	−1.00	−0.05	−0.16	0.16	0.30
SES	−0.01	−0.27	0.11	−0.10	0.19	0.26
Race: Black	−0.23	0.07	0.01	−0.03	0.06	−0.1
Race: Asian	−0.13	0.51	−0.04	−0.16	0.26	−0.12
Race: biracial	−0.10	−1.24	0.22	0.11	−0.07	0.07
Race: other	−0.17	1.50	0.01	−0.06	−0.06	0.04
R2	57.1%	43.1%	73.7%	49.1%	63.8%	44.6%

* indicates *p* < 0.05; ** indicates *p* < 0.01; *** indicates *p* < 0.001; FGCS = first-generation college students; reference group: control group, male, non-FGCS, white. Standardized coefficients were reported.

## Data Availability

Not applicable.
